# 3-Isobutyl 5-methyl 2,6-dimethyl-4-(2-nitro­phen­yl)pyridine-3,5-dicarboxyl­ate

**DOI:** 10.1107/S1600536809051988

**Published:** 2009-12-09

**Authors:** Hui Chen, Jing Luo, Lin-Lin Jing, Ru Jiang

**Affiliations:** a Department of Chemistry, School of Pharmacy, Fourth Military Medical University, Changle West Road 17, 710032, Xi-An, People’s Republic of China; b Department of Shannxi Institute for Food and Drug Control, Zhuque Road 431,710061 Xi-An, People’s Republic of China

## Abstract

The title nitro­phenyl pyridine compound, C_20_H_22_N_2_O_6_ was synthesized as a degradation product of the hypertension medication nisoldipine. The dihedral angle between the nitro-substituted phenyl ring and the pyridine ring is 75.5 (4)°. There are a number of C—H⋯O inter­actions between symmetry-related mol­ecules>.

## Related literature

For the preparation of the title compound see: Agbaba *et al.* (2004 ▶); Waldo & Correa (2001 ▶); Valentina *et al.* (2000 ▶). A derivative of the title compound, nisoldipine, has been evaluated as a calcium channel blocker with vasodilator properties, see: Ferrari *et al.* (2005 ▶); Marciniec *et al.* (2002 ▶); Kazda *et al.* (1980 ▶).
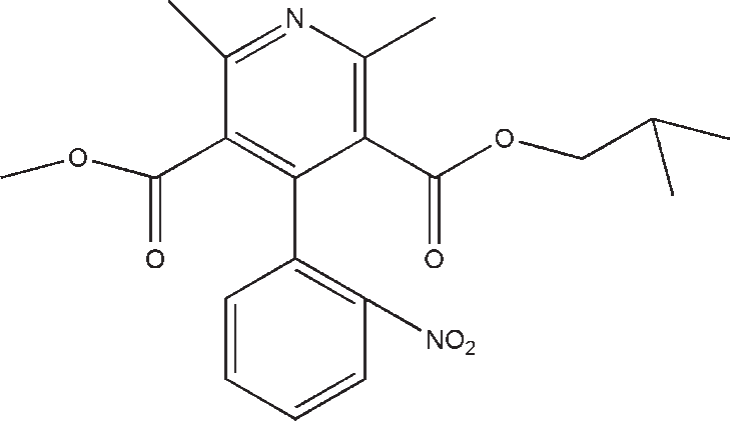


## Experimental

### 

#### Crystal data

C_20_H_22_N_2_O_6_
*M* *_r_* = 386.40
Monoclinic, 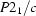

*a* = 8.4222 (9) Å
*b* = 16.5850 (16) Å
*c* = 14.5011 (15) Å
β = 102.748 (2)°
*V* = 1975.6 (4) Å^3^
*Z* = 4
Mo *K*α radiation
μ = 0.10 mm^−1^
*T* = 296 K
0.12 × 0.10 × 0.08 mm

#### Data collection

Bruker APEXII CCD diffractometer
Absorption correction: multi-scan (*SADABS*; Bruker, 2001 ▶) *T*_min_ = 0.989, *T*_max_ = 0.992
10199 measured reflections
3683 independent reflections
2718 reflections with *I* > 2σ(*I*)
*R*_int_ = 0.020

#### Refinement

*R*[*F* ^2^ > 2σ(*F* ^2^)] = 0.046
*wR*(*F* ^2^) = 0.154
*S* = 1.01
3683 reflections
259 parameters
H-atom parameters constrained
Δρ_max_ = 0.28 e Å^−3^
Δρ_min_ = −0.21 e Å^−3^

### 

Data collection: *APEX2* (Bruker, 2004 ▶); cell refinement: *SAINT-Plus* (Bruker, 2001 ▶); data reduction: *SAINT-Plus*; program(s) used to solve structure: *SHELXS97* (Sheldrick, 2008 ▶); program(s) used to refine structure: *SHELXL97* (Sheldrick, 2008 ▶); molecular graphics: *SHELXTL* (Sheldrick, 2008 ▶); software used to prepare material for publication: *SHELXTL*.

## Supplementary Material

Crystal structure: contains datablocks I. DOI: 10.1107/S1600536809051988/pk2213sup1.cif

Structure factors: contains datablocks I. DOI: 10.1107/S1600536809051988/pk2213Isup2.hkl

Additional supplementary materials:  crystallographic information; 3D view; checkCIF report

## Figures and Tables

**Table 1:** Hydrogen-bond geometry (Å, °)

*D*—H⋯*A*	*D*—H	H⋯*A*	*D*⋯*A*	*D*—H⋯*A*
C12—H12*C*⋯O4^i^	0.96	2.57	3.303 (3)	134
C13—H13*B*⋯O1^ii^	0.96	2.48	3.395 (3)	160
C14—H14*B*⋯O6^iii^	0.96	2.52	3.221 (3)	130

Symmetry codes: (i) 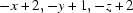; (ii) 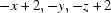; (iii) 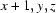.

**Fig. 1.**
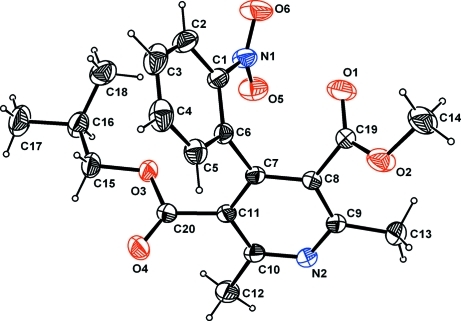
Molecular structure of the title compound. Displacement ellipsoids are drawn at the 30% probability level.

## supplementary crystallographic information

### Comment

Nisoldipine is a calcium channel blocker with vasodilator properties (Marciniec *et al. *2002). It is used in several commercial preparations for treatment of hypertension (Kazda *et al. *1980). It has been the subject of many analytical chemical investigations. Synthetic conditions result in side-reactions and formation of 1,4-dihydropyridines. This compound is a degradation product of nisoldipine.

The molecular structure is shown in Fig 1.  The dihedral angle between the planes of phenyl and the pyridyl rings is 75.5 (4)°. There are a number of C—H···O interactions between symmetry-related molecules.

### Experimental

A solution of nisoldipine (10 mmol) in 50 mL anhydrous ethanol was exposed to sunlight for 10 h at ambient temperature.  50 mL of water was added to the mixture and it was then filtered.  The crude product was purified by flash chromatography (40/60 ethyl acetate/ether).

### Refinement

H-atoms were included in calculated positions and treated as riding atoms: C—H = 0.92—0.96 Å with with *U*_iso_(H) = 1.2*U*_eq_(C) or 1.5 *U*_eq_(C_Me_)

### Crystal data

**Table:**

C_20_H_22_N_2_O_6_	*F*(000) = 816
*M**_r_* = 386.40	*D*_x_ = 1.299 Mg m^−^^3^
Monoclinic, *P*2_1_/*c*	Mo *K*α radiation, λ = 0.71073 Å
Hall symbol: -P 2ybc	Cell parameters from 3360 reflections
*a* = 8.4222 (9) Å	θ = 2.5–25.3°
*b* = 16.5850 (16) Å	µ = 0.10 mm^−^^1^
*c* = 14.5011 (15) Å	*T* = 296 K
β = 102.748 (2)°	Block, colorless
*V* = 1975.6 (4) Å^3^	0.12 × 0.10 × 0.08 mm
*Z* = 4	

### Data collection

**Table:**

Bruker APEXII CCD diffractometer	3683 independent reflections
Radiation source: fine-focus sealed tube	2718 reflections with *I* > 2σ(*I*)
graphite	*R*_int_ = 0.020
φ and ω scans	θ_max_ = 25.5°, θ_min_ = 1.9°
Absorption correction: multi-scan (*SADABS*; Bruker, 2001)	*h* = −9→10
*T*_min_ = 0.989, *T*_max_ = 0.992	*k* = −20→16
10199 measured reflections	*l* = −17→14

### Refinement

**Table:**

Refinement on *F*^2^	Primary atom site location: structure-invariant direct methods
Least-squares matrix: full	Secondary atom site location: difference Fourier map
*R*[*F*^2^ > 2σ(*F*^2^)] = 0.046	Hydrogen site location: inferred from neighbouring sites
*wR*(*F*^2^) = 0.154	H-atom parameters constrained
*S* = 1.01	*w* = 1/[σ^2^(*F*_o_^2^) + (0.090*P*)^2^ + 0.3869*P*] where *P* = (*F*_o_^2^ + 2*F*_c_^2^)/3
3683 reflections	(Δ/σ)_max_ < 0.001
259 parameters	Δρ_max_ = 0.28 e Å^−^^3^
0 restraints	Δρ_min_ = −0.21 e Å^−^^3^

### Special details

**Table:**

Geometry. All e.s.d.'s (except the e.s.d. in the dihedral angle between two l.s. planes) are estimated using the full covariance matrix. The cell e.s.d.'s are taken into account individually in the estimation of e.s.d.'s in distances, angles and torsion angles; correlations between e.s.d.'s in cell parameters are only used when they are defined by crystal symmetry. An approximate (isotropic) treatment of cell e.s.d.'s is used for estimating e.s.d.'s involving l.s. planes.
Refinement. Refinement of *F*^2^ against ALL reflections. The weighted *R*-factor *wR* and goodness of fit *S* are based on *F*^2^, conventional *R*-factors *R* are based on *F*, with *F* set to zero for negative *F*^2^. The threshold expression of *F*^2^ > σ(*F*^2^) is used only for calculating *R*-factors(gt) *etc*. and is not relevant to the choice of reflections for refinement. *R*-factors based on *F*^2^ are statistically about twice as large as those based on *F*, and *R*– factors based on ALL data will be even larger.

### Fractional atomic coordinates and isotropic or equivalent isotropic displacement parameters (Å^2^)

**Table:**

	*x*	*y*	*z*	*U*_iso_*/*U*_eq_	
C1	0.6743 (2)	0.17497 (11)	0.80315 (14)	0.0478 (5)	
C2	0.5729 (3)	0.17694 (15)	0.71437 (18)	0.0685 (7)	
H2	0.4764	0.1476	0.7020	0.082*	
C3	0.6151 (4)	0.22258 (17)	0.64421 (17)	0.0765 (8)	
H3	0.5462	0.2250	0.5846	0.092*	
C4	0.7583 (3)	0.26433 (15)	0.66227 (16)	0.0700 (7)	
H4	0.7877	0.2944	0.6145	0.084*	
C5	0.8585 (3)	0.26212 (12)	0.75035 (15)	0.0546 (5)	
H5	0.9559	0.2907	0.7614	0.066*	
C6	0.8188 (2)	0.21826 (11)	0.82405 (13)	0.0412 (4)	
C7	0.9348 (2)	0.22295 (10)	0.91819 (12)	0.0376 (4)	
C8	1.0480 (2)	0.16259 (10)	0.95138 (13)	0.0403 (4)	
C9	1.1657 (2)	0.17571 (11)	1.03476 (14)	0.0443 (5)	
C10	1.0622 (2)	0.30249 (11)	1.05295 (14)	0.0457 (5)	
C11	0.9444 (2)	0.29486 (10)	0.96861 (13)	0.0396 (4)	
C12	1.0756 (3)	0.37681 (14)	1.11321 (18)	0.0692 (7)	
H12A	1.1441	0.3659	1.1741	0.104*	
H12B	0.9693	0.3923	1.1206	0.104*	
H12C	1.1219	0.4198	1.0834	0.104*	
C13	1.2955 (3)	0.11554 (13)	1.07470 (17)	0.0617 (6)	
H13A	1.3734	0.1132	1.0354	0.093*	
H13B	1.2471	0.0634	1.0768	0.093*	
H13C	1.3492	0.1315	1.1375	0.093*	
C14	1.1842 (3)	−0.01320 (15)	0.8348 (2)	0.0854 (9)	
H14A	1.1540	−0.0537	0.8750	0.128*	
H14B	1.2928	−0.0234	0.8272	0.128*	
H14C	1.1102	−0.0147	0.7741	0.128*	
C15	0.5685 (2)	0.41210 (14)	0.8841 (2)	0.0660 (6)	
H15A	0.5611	0.4127	0.8164	0.079*	
H15B	0.6066	0.4646	0.9092	0.079*	
C16	0.4058 (3)	0.39528 (15)	0.90375 (19)	0.0657 (6)	
H16	0.4179	0.3963	0.9725	0.079*	
C17	0.2888 (3)	0.46254 (19)	0.8627 (2)	0.0936 (9)	
H17A	0.3328	0.5134	0.8878	0.140*	
H17B	0.1859	0.4537	0.8792	0.140*	
H17C	0.2738	0.4630	0.7951	0.140*	
C18	0.3416 (3)	0.31309 (17)	0.8696 (2)	0.0772 (7)	
H18A	0.3292	0.3100	0.8023	0.116*	
H18B	0.2380	0.3046	0.8853	0.116*	
H18C	0.4167	0.2724	0.8994	0.116*	
C19	1.0395 (2)	0.08501 (11)	0.89873 (15)	0.0471 (5)	
C20	0.8372 (2)	0.36417 (11)	0.93117 (14)	0.0445 (4)	
N1	0.62323 (19)	0.12682 (11)	0.87582 (15)	0.0588 (5)	
N2	1.16919 (19)	0.24420 (10)	1.08444 (12)	0.0492 (4)	
O1	0.9204 (2)	0.04564 (10)	0.87632 (16)	0.0907 (7)	
O2	1.17751 (19)	0.06580 (9)	0.87727 (13)	0.0710 (5)	
O3	0.68230 (15)	0.34992 (8)	0.92811 (11)	0.0558 (4)	
O4	0.88648 (19)	0.42597 (9)	0.90518 (15)	0.0768 (5)	
O5	0.6685 (2)	0.14682 (11)	0.95800 (12)	0.0725 (5)	
O6	0.5362 (2)	0.06843 (12)	0.85059 (16)	0.0947 (6)	

### Atomic displacement parameters (Å^2^)

**Table:**

	*U*^11^	*U*^22^	*U*^33^	*U*^12^	*U*^13^	*U*^23^
C1	0.0388 (10)	0.0479 (11)	0.0536 (11)	0.0019 (8)	0.0038 (8)	−0.0091 (9)
C2	0.0533 (13)	0.0721 (15)	0.0683 (16)	0.0055 (11)	−0.0123 (11)	−0.0226 (13)
C3	0.092 (2)	0.0798 (17)	0.0451 (13)	0.0281 (15)	−0.0131 (12)	−0.0107 (12)
C4	0.0932 (19)	0.0689 (15)	0.0452 (13)	0.0206 (14)	0.0095 (12)	0.0037 (11)
C5	0.0598 (13)	0.0534 (12)	0.0503 (12)	0.0046 (9)	0.0114 (10)	0.0032 (9)
C6	0.0401 (9)	0.0398 (9)	0.0419 (10)	0.0058 (7)	0.0055 (8)	−0.0032 (8)
C7	0.0324 (9)	0.0385 (9)	0.0416 (10)	−0.0045 (7)	0.0076 (7)	0.0009 (7)
C8	0.0373 (9)	0.0349 (9)	0.0481 (10)	−0.0035 (7)	0.0079 (8)	0.0025 (8)
C9	0.0385 (9)	0.0405 (10)	0.0516 (11)	−0.0036 (7)	0.0048 (8)	0.0042 (8)
C10	0.0406 (10)	0.0438 (10)	0.0505 (11)	−0.0046 (8)	0.0050 (8)	−0.0062 (9)
C11	0.0322 (9)	0.0383 (9)	0.0489 (10)	−0.0028 (7)	0.0100 (8)	−0.0006 (8)
C12	0.0661 (14)	0.0570 (14)	0.0759 (16)	−0.0003 (11)	−0.0032 (12)	−0.0225 (12)
C13	0.0572 (13)	0.0538 (13)	0.0644 (14)	0.0057 (10)	−0.0074 (10)	0.0045 (10)
C14	0.0797 (17)	0.0593 (15)	0.121 (2)	0.0004 (13)	0.0313 (16)	−0.0361 (15)
C15	0.0462 (12)	0.0578 (13)	0.0916 (17)	0.0121 (10)	0.0101 (11)	0.0198 (12)
C16	0.0487 (12)	0.0741 (15)	0.0734 (15)	0.0114 (11)	0.0116 (11)	0.0161 (12)
C17	0.0564 (15)	0.098 (2)	0.124 (3)	0.0277 (14)	0.0160 (15)	0.0310 (19)
C18	0.0513 (13)	0.0931 (19)	0.0862 (18)	−0.0068 (13)	0.0130 (12)	0.0095 (15)
C19	0.0409 (10)	0.0378 (10)	0.0592 (12)	−0.0002 (8)	0.0035 (9)	0.0003 (9)
C20	0.0402 (10)	0.0391 (10)	0.0533 (11)	−0.0018 (8)	0.0084 (8)	−0.0020 (8)
N1	0.0403 (9)	0.0596 (11)	0.0751 (13)	−0.0099 (8)	0.0099 (9)	−0.0080 (10)
N2	0.0433 (9)	0.0479 (9)	0.0512 (10)	−0.0021 (7)	−0.0010 (7)	−0.0025 (7)
O1	0.0535 (9)	0.0594 (10)	0.1577 (19)	−0.0133 (8)	0.0201 (11)	−0.0422 (11)
O2	0.0602 (9)	0.0568 (9)	0.1024 (13)	−0.0086 (7)	0.0317 (9)	−0.0292 (8)
O3	0.0370 (7)	0.0486 (8)	0.0804 (10)	0.0036 (6)	0.0101 (7)	0.0143 (7)
O4	0.0538 (9)	0.0472 (9)	0.1299 (16)	0.0000 (7)	0.0214 (9)	0.0233 (9)
O5	0.0717 (11)	0.0852 (12)	0.0627 (11)	−0.0205 (9)	0.0197 (8)	−0.0001 (9)
O6	0.0713 (11)	0.0836 (13)	0.1256 (17)	−0.0391 (10)	0.0141 (11)	−0.0118 (11)

### Geometric parameters (Å, °)

**Table:**

C1—C2	1.379 (3)	C13—H13B	0.9600
C1—C6	1.388 (3)	C13—H13C	0.9600
C1—N1	1.460 (3)	C14—O2	1.454 (3)
C2—C3	1.376 (4)	C14—H14A	0.9600
C2—H2	0.9300	C14—H14B	0.9600
C3—C4	1.365 (4)	C14—H14C	0.9600
C3—H3	0.9300	C15—O3	1.456 (2)
C4—C5	1.367 (3)	C15—C16	1.486 (3)
C4—H4	0.9300	C15—H15A	0.9700
C5—C6	1.393 (3)	C15—H15B	0.9700
C5—H5	0.9300	C16—C18	1.509 (4)
C6—C7	1.495 (2)	C16—C17	1.521 (3)
C7—C11	1.392 (2)	C16—H16	0.9800
C7—C8	1.393 (2)	C17—H17A	0.9600
C8—C9	1.401 (3)	C17—H17B	0.9600
C8—C19	1.490 (3)	C17—H17C	0.9600
C9—N2	1.342 (2)	C18—H18A	0.9600
C9—C13	1.499 (3)	C18—H18B	0.9600
C10—N2	1.332 (2)	C18—H18C	0.9600
C10—C11	1.400 (3)	C19—O1	1.181 (2)
C10—C12	1.501 (3)	C19—O2	1.307 (2)
C11—C20	1.488 (3)	C20—O4	1.197 (2)
C12—H12A	0.9600	C20—O3	1.317 (2)
C12—H12B	0.9600	N1—O6	1.221 (2)
C12—H12C	0.9600	N1—O5	1.214 (2)
C13—H13A	0.9600		
			
C2—C1—C6	121.6 (2)	H13B—C13—H13C	109.5
C2—C1—N1	117.79 (19)	O2—C14—H14A	109.5
C6—C1—N1	120.59 (17)	O2—C14—H14B	109.5
C3—C2—C1	119.7 (2)	H14A—C14—H14B	109.5
C3—C2—H2	120.2	O2—C14—H14C	109.5
C1—C2—H2	120.2	H14A—C14—H14C	109.5
C4—C3—C2	119.9 (2)	H14B—C14—H14C	109.5
C4—C3—H3	120.0	O3—C15—C16	109.20 (18)
C2—C3—H3	120.0	O3—C15—H15A	109.8
C3—C4—C5	120.2 (2)	C16—C15—H15A	109.8
C3—C4—H4	119.9	O3—C15—H15B	109.8
C5—C4—H4	119.9	C16—C15—H15B	109.8
C4—C5—C6	121.8 (2)	H15A—C15—H15B	108.3
C4—C5—H5	119.1	C15—C16—C18	112.7 (2)
C6—C5—H5	119.1	C15—C16—C17	109.4 (2)
C1—C6—C5	116.75 (18)	C18—C16—C17	112.3 (2)
C1—C6—C7	126.24 (17)	C15—C16—H16	107.4
C5—C6—C7	117.00 (17)	C18—C16—H16	107.4
C11—C7—C8	118.55 (16)	C17—C16—H16	107.4
C11—C7—C6	118.36 (15)	C16—C17—H17A	109.5
C8—C7—C6	122.52 (15)	C16—C17—H17B	109.5
C7—C8—C9	119.11 (16)	H17A—C17—H17B	109.5
C7—C8—C19	119.47 (16)	C16—C17—H17C	109.5
C9—C8—C19	121.41 (16)	H17A—C17—H17C	109.5
N2—C9—C8	121.50 (16)	H17B—C17—H17C	109.5
N2—C9—C13	115.35 (17)	C16—C18—H18A	109.5
C8—C9—C13	123.15 (17)	C16—C18—H18B	109.5
N2—C10—C11	121.95 (17)	H18A—C18—H18B	109.5
N2—C10—C12	116.00 (17)	C16—C18—H18C	109.5
C11—C10—C12	122.05 (17)	H18A—C18—H18C	109.5
C7—C11—C10	118.98 (16)	H18B—C18—H18C	109.5
C7—C11—C20	120.65 (16)	O1—C19—O2	123.08 (19)
C10—C11—C20	120.30 (16)	O1—C19—C8	124.05 (19)
C10—C12—H12A	109.5	O2—C19—C8	112.84 (16)
C10—C12—H12B	109.5	O4—C20—O3	123.53 (18)
H12A—C12—H12B	109.5	O4—C20—C11	123.49 (17)
C10—C12—H12C	109.5	O3—C20—C11	112.97 (15)
H12A—C12—H12C	109.5	O6—N1—O5	123.3 (2)
H12B—C12—H12C	109.5	O6—N1—C1	118.1 (2)
C9—C13—H13A	109.5	O5—N1—C1	118.68 (17)
C9—C13—H13B	109.5	C10—N2—C9	119.82 (16)
H13A—C13—H13B	109.5	C19—O2—C14	116.10 (18)
C9—C13—H13C	109.5	C20—O3—C15	116.05 (15)
H13A—C13—H13C	109.5		

### Hydrogen-bond geometry (Å, °)

**Table:**

*D*—H···*A*	*D*—H	H···*A*	*D*···*A*	*D*—H···*A*
C12—H12C···O4^i^	0.96	2.57	3.303 (3)	134
C13—H13B···O1^ii^	0.96	2.48	3.395 (3)	160
C14—H14B···O6^iii^	0.96	2.52	3.221 (3)	130

Symmetry codes: (i) −*x*+2, −*y*+1, −*z*+2; (ii) −*x*+2, −*y*, −*z*+2; (iii) *x*+1, *y*, *z*.
